# Characterization of Steam Volatiles and Evaluation of the Antioxidant Properties of Different Extracts from Leaves and Roots of *Aegopodium podagraria* L.

**DOI:** 10.3390/molecules30244786

**Published:** 2025-12-15

**Authors:** Renata Baranauskienė, Ieva Račkauskienė, Petras Rimantas Venskutonis

**Affiliations:** Department of Food Science and Technology, Kaunas University of Technology, Radvilėnų rd. 19, LT-50254 Kaunas, Lithuania; renata.baranauskiene@ktu.lt (R.B.); ieva.rackauskiene@gmail.com (I.R.)

**Keywords:** *Aegopodium podagraria* L., goutweed, ground elder, essential oil, water extract, methanol extract, acetone extract, antioxidant capacity

## Abstract

This study presents the results on the composition of hydro-distilled essential oils and the antioxidant properties of extracts isolated with different polarity solvents from the leaves (GLEO) and roots (GREO) of wild-grown Lithuanian goutweed (*Aegopodium podagraria* L.). The yields of GLEO and GREO were 0.22% and 0.04%. The identified compounds numbered 117 and 88, which constituted 99.4 and 99.2% of the total integrated peak area, respectively. The major GLEO constituents were sesquiterpenes germacrene D (17.53%), (*E*)-β-bergamotene (11.75%), (*E*,*E*)-α-farnesene (7.23%), and (*E*)-caryophyllene (5.29%), while monoterpene α-pinene (19.24%) was quantitatively dominant in GREO, followed by sesquiterpenes germacrene B (4.59%), (*E*)-caryophyllene (4.51%), β-barbatene (4.26%), and β-bazzanene (4.10%). Polyacetylene (*Z*)-falcarinol, which is an important bioactive compound, constituted 4.60% in GREO. The antioxidant characteristics of water, methanol, and acetone extracts were evaluated by the TPC, DPPH^•^/ABTS^•+^ scavenging, and ORAC assays. The water and methanol extracts of the leaves were the strongest antioxidants; their TPC and ORAC values were 62.12 and 56.84 mg GAE/g, and 1426 and 1293 µM TE/g, respectively; the EC_50_ values of DPPH^•^ and ABTS^•+^ scavenging were 1.18 and 2.48, and 2.45 and 3.57 mg/mL, respectively. The results obtained may assist in developing antioxidants, cosmeceuticals, nutraceuticals, and other health-beneficial preparations from *A. podagraria* extracts.

## 1. Introduction

*Aegopodium podagraria* L. (goutweed, ground elder) belongs to the carrot family, Apiaceae. The plant is spread throughout Europe, Asia, and North America, and is considered a “common weed”. It is widely distributed in shady, humid places and forms dense vegetation in deciduous forests, woodlands, bushes, clearings, fences, gardens, and parks. However, it is an edible plant: young leaves of goutweed can be used in soups, salads, in the spring, and in feed formulations as well [1,2].

*A. podagraria* has been traditionally used in Europe in folk and monastic medicine for gout and similar ailments, known as podagra, as well as joint pains [3] and arthritis [4]. For many years, *A. podagraria* has been used in traditional medicine, primarily to treat urinary and kidney diseases [5,6]. Recently, it has also been found to have potential benefits in the treatment of prostate cancer [7]. The health benefits of goutweed have been reported by many studies since the beginning of the 20th century. The chemical composition of *A. podagraria* shows that this plant is nutritious and rich in health-promoting compounds, making it a valuable addition to medicinal practices [1,2,3,4,8,9,10,11,12,13].

Goutweed leaves, stems, and flowers contain essential oils (EOs), flavonoids, nitrogen compounds, free amino acids, steroids, vitamins C and E, microelements, enzymes, and phytoncides; the roots are also rich in carbohydrates, proteins, nitrogen, and other organic compounds, as well as coumarins, phenols, and carboxylic acids [2,4,10,14]. The rhizomes of goutweed have been found to contain glycoproteins that bind carbohydrates, mainly glycoprotein lectin [15]. Polyphenols contributing to the antioxidative potential of this plant are phenolic acids (caffeic, ferulic, and chlorogenic), coumarins, flavonoids, and especially aliphatic C_17_-polyacetylenes [2,13]. Polyacetylenes (polyins), including falcarinol, falcarindiol, falcarinon, and falcarinolon, are among the most important bioactive compounds occurring in plants of the Apiaceae family [11]. Falcarinol possesses anti-inflammatory and anticoagulant properties by influencing prostaglandin metabolism [1] and is toxic against fungi, bacteria, viral infection agents, and mammalian cells [2]. EO is another important group of secondary metabolites of goutweed; it consists mainly of mono- and sesquiterpenes [1,2,4,5,16,17]. EOs, as a source of naturally occurring phytochemicals, are well known not only for their antimicrobial propensities [18], but also for their psychotherapeutic effects [19], making them potential alternatives to antibiotics and conventional medications for the treatment of anxiety and depression. Lipophilic antioxidants such as carotenoids, chlorophylls, and tocopherols have been reported to be present in comparatively high amounts in the leaves; lutein, neoxanthin, and α-tocopherol were the major compounds found from these groups [20]. Coumarins were also reported to be an essential group of goutweed’s bioactive compounds, including arterin (roots), angular furanocoumarins (leaves), and angelicin (leaves, fruits, and roots) [14]. The microelements such as potassium, chromium, zinc, copper, and manganese were among the significant elements in *A. podagraria*‘s leaves and stems [2,4].

It was reported that goutweed tinctures and extracts can be used to treat gout and metabolic diseases, including type 2 diabetes [14]. Nizioł-Łukaszewska et al. [12] suggested the potential of using *A. podagraria* in cosmetic and pharmaceutical preparations, as it demonstrated positive effects on the skin cells; *A. podagraria* extracts suppressed the activity of elastase and collagenase and stimulated the migration of keratinocytes and fibroblasts on model skin cells. The potential of this plant for drug development was highlighted by Prior et al. [13]. Leaves, roots, and flowers of *A. podagraria* inhibited cyclooxygenase-1 (COX-1) in vitro, and this effect was associated with polyacetylene falcarindiol, which was present in all plant parts and is responsible for anti-inflammatory and antimicrobial properties [13]. The antibacterial activity of an ethanol extract of goutweed was shown by Stefanovic et al. [10]. The antiviral activity in vitro of ethanol and aqueous extracts in an MDCK cell culture against the H3N2 and H5N1 subtypes of influenza A virus (IAV) was reported by Mazurkova et al. [9]. *A. podagraria* flower-derived EO possessed diuretic and uricosuric pharmacological activity in a dose of 1 mL/kg [5]. In general, the pharmacological activities of *A. podagraria* preparations are associated with hydroxycinnamic acids, flavonoids, coumarins, polyacetylenes, and micro- and macro-elements, and other bioactive compounds present in goutweed.

This study aimed to evaluate the phytochemical composition and antioxidant characteristics of *A. podagraria* using a more systematic approach, which has not been previously applied to this plant and may increase the sustainability of its processing. The main objectives were to determine the chemical composition of its EOs, to determine its total content of polyphenolics, and to evaluate the oxygen radical absorbance and radical scavenging capacities of extracts isolated from the leaves and roots of *A. podagraria* growing wild in Lithuania. To the best of our knowledge, this is the first report on goutweed to provide more comprehensive insights into its potential uses as a source of bioactive compounds.

## 2. Results and Discussion

### 2.1. The Composition of Essential Oils (EOs)

The yield and chemical composition of EOs depend on climatic conditions, growing site, harvesting time, country of origin, post-harvesting handling, processing procedures, and other factors [2,4,5,16,21,22]. The yield of EO isolated from goutweed leaves was 0.22 ± 0.01%, whereas that from the roots was very low, approximately 0.04%. Information on EO yields from goutweed is relatively scarce. EO yield was quite similar to that obtained from growing wild goutweed in Estonia: 1.7 and 3.8 mg/g from the leaves and stems, respectively [2]. A similar yield (0.22%) was obtained from goutweed flowers collected in the Kharkiv region of Ukraine [5]. The EO from *A. podagraria* from Kopaonik (Central Balkan) yielded 0.11% [16].

**Figure 1:**
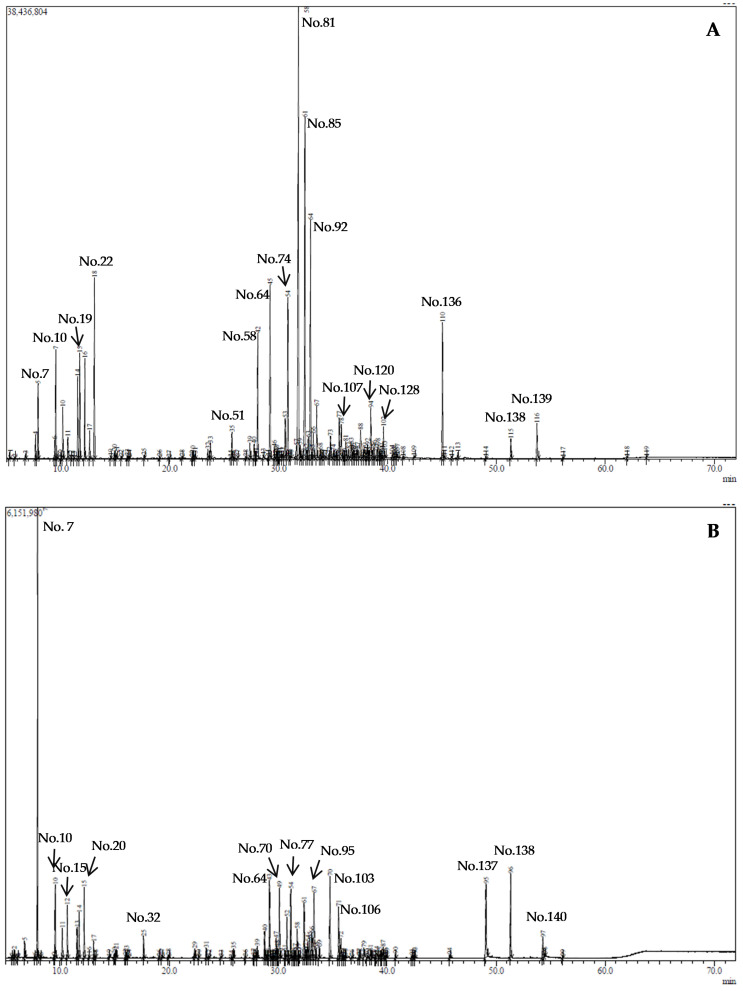
The GC-MS chromatograms of *Aegopodium podagraria* GLEO (**A**) and GREO (**B**) (The number of the major peaks marked as No. corresponds to the number of compounds presented in Table 1).

The detailed list of *A. podagraria* EOs volatile compounds, their percentage composition, as well as their odour descriptors, are presented in Table 1, and the representative GC-MS chromatograms are presented in Figure 1. In total, 144 different compounds were identified in the EOs from the leaves (117) and roots (88); they accounted for 99.4% and 99.2% of the total integrated GC peak area, respectively. To the best of our knowledge, 58 compounds have not been reported in *A. podagraria* previously.

It is evident that the EOs of *A. podagraria* grown in Lithuania consist mostly of mono- and sesquiterpene hydrocarbons, and this agrees with other studies [1,2,5,16,17]. The major GLEO constituents were sesquiterpenes germacrene D (17.53 ± 0.13%), (*E*)-β-bergamotene (11.75 ± 0.02%), (*E*,*E*)-α-farnesene (7.23 ± 0.03%), (*E*)-caryophyllene (5.29 ± 0.02%), (*E*)-β-farnesene (4.83 ± 0.01%), and β-elemene (3.71 ± 0.03%); the major monoterpenes were γ-terpinene (4.54 ± 0.04%), β-pinene (2.42 ± 0.02%), limonene (2.45 ± 0.03%), and (*Z*)-β-ocimene (2.29 ± 0.01%) (Table 1). Higher diterpene alcohols, such as isophytol and (*E*)-phytol, were identified in GLEO and accounted for 4.06 ± 0.01% and 1.15 ± 0.06%, respectively; they are used in the flavour industry and also serve as precursors in the production of vitamins K and E.

To the best of our knowledge, the composition of *A. podagraria* roots has not been reported previously. The principal constituent, quantitatively, of GREO was monoterpene α-pinene (19.24 ± 0.09%), followed by β-pinene (3.22 ± 0.02%), limonene (2.04 ± 0.01%), (*Z*)-β-ocimene (3.11 ± 0.01%), and (*E*)-β-farnesesne (2.32 ± 0.03%). The major sesquiterpene hydrocarbons were germacrene B (4.59 ± 0.03%), (*E*)-caryophyllene (4.51 ± 0.03%), β-barbatene (4.26 ± 0.04%), β-bazzanene (4.10 ± 0.32%), β-acoradiene (3.77 ± 0.06%), and bicyclogermacrene (3.33 ± 0.01%), while spathulenol (2.94 ± 0.04%) was the dominant oxygenated sesquiterpene (Table 1). The contents of hexadecanoic and linoleic fatty acids were 4.89 ± 0.06% and 1.37 ± 0.11%, respectively, while *n*-octanal (2.35 ± 0.02%) was the major aldehyde. It may be observed that the compositional differences between GREO and GLEO were quite significant.

Another quantitatively important bioactive constituent determined in GREO was polyacetylene (*Z*)-falcarinol (4.60 ± 0.01%). The content of sesquiterpene hydrocarbon cuparene (0.89 ± 0.01%) and phenylpropanoid myristicin (0.61 ± 0.01%) was lower than 1%. Polyacetylenic oxylipin falcarinol was reported to possess antitumor, insecticidal, and other bioactivities [23], and it was highly toxic against bacteria, fungi, viral infection agents, and mammalian cells [2]. Myristicin is a major bioactive component in nutmeg EO and exhibits antioxidant, analgesic, anti-inflammatory, insecticidal, and anticancer activities [24].

**Table 1:** Chemical composition of essential oils of *Aegopodium podagraria* L. isolated from leaves and roots, peak area percentage.

No #	Compound ^A^	KI Calc. ^B^	KI Lit. ^C^	GLEO	GREO	Odour Description
1	(*E*)-2-Hexenal	847	855	0.14 ± 0.00 ^E^		green, leaf ^1^; sharp, fresh, leafy, green, clean, fruity, herbal, spicy, herbal ^2^
2	(*2E*)-Hexenol *	866	862		tr ^F^	green, leaf, walnut ^1^; fresh, fatty, green, fruity, vegetable, leafy, herbal ^2^
3	*n*-Hexanol	869	870	tr	0.32 ± 0.02	resin, flower, green ^1^; ethereal, fusel, oily, fruity, alcoholic, sweet, green ^2^
4	*n*-Nonane	900	900	tr	0.98 ± 0.01	alkane ^1^
5	Heptanal	901	902	0.12 ± 0.00		fat, citrus, rancid ^1^; fresh, aldehydic, fatty, green, herbal, cognac, ozone ^2^
6	α-Thujene	929	930	0.48 ± 0.01	0.07 ± 0.00	wood, green, herb ^1^; woody, green, herbal ^2^
7	α-Pinene	936	939	1.60 ± 0.02	19.24 ± 0.09	pine, turpentine^1^; fresh, camphoreous, sweet, pine, earthy, woody ^2^
8	Camphene	954	954	tr		camphor ^1^; woody, herbal, fir, needle, camphoreous, terpenic ^2^
9	Sabinene	975	975	0.42 ± 0.01	0.10 ± 0.01	pepper, turpentine, wood ^1^; wood, spicy, citrus, terpenic, green, oily, camphoreous ^2^
10	β-Pinene	978	979	2.42 ± 0.02	3.22 ± 0.02	pine, resin, turpentine ^1^; dry, woody, resinous, pine, hay, green, eucalyptus, camphoreous ^2^
11	1-Octen-3-ol	981	979	tr		mushroom ^1^
12	6-Methyl-5-hepten-2-one	988	985	0.12 ± 0.00		citrus, green, musty, lemongrass, apple ^2^
13	Myrcene	992	990	1.14 ± 0.02	1.33 ± 0.01	balsamic, must, spice ^1^; terpenic, herbal, woody, rose, celery, carrot ^2^
14	3-Octanol	992	991	0.05 ± 0.00		moss, nut, mushroom ^1^; earthy, mushroom, herbal, melon, citrus, woody, spicy, minty ^2^
15	*n*-Octanal	1003	998	0.62 ± 0.01	2.35 ± 0.02	fat, soap, lemon, green ^1^; aldehydic, waxy, citrus, orange, peel, green, herbal, fresh, fatty ^2^
16	(*2E*,*4E*)-Heptadienal *	1010	1007	tr		nut, fat ^1^; fatty, green, oily, aldehydic, vegetable ^2^
17	α-Terpinene	1018	1017	tr		lemon ^1^; woody, terpenic, lemon, herbal, medicinal, citrus ^2^
18	*p*-Cymene	1026	1024	1.93 ± 0.01	1.33 ± 0.01	solvent, gasoline, citrus ^1^; fresh, citrus, terpenic, woody, spicy ^2^
19	Limonene	1031	1029	2.45 ± 0.03	2.04 ± 0.01	lemon, orange ^1^; citrus, orange, fresh, sweet ^2^
20	(*Z*)-β-Ocimene	1042	1037	2.29 ± 0.01	3.11 ± 0.01	citrus, herb, flower ^1^; warm, floral, herbal, sweet ^2^
21	(*E*)-β-Ocimene	1052	1050	0.65 ± 0.01	0.16 ± 0.01	sweet, herb ^1^
22	γ-Terpinene	1062	1059	4.54 ± 0.04	0.77 ± 0.03	gasoline, turpentine ^1^; oily, woody, terpenic, lemon, lime, tropical, herbal ^2^
23	Terpinolene	1090	1088	0.10 ± 0.02	0.05 ± 0.01	fresh, woody, sweet, pine, citrus ^2^
24	2-Nonanone	1093	1090	0.06 ± 0.00	0.11 ± 0.01	hot milk, soap, green ^1^; fresh, sweet, green, weedy, earthy, herbal ^2^
25	Linalool	1099	1096	0.19 ± 0.00	tr	flower, lavender ^1^; citrus, orange, floral, terpenic, waxy, rose ^2^
26	*n*-Undecane *	1100	1100		0.23 ± 0.01	alkane ^1^
27	*n*-Nonanal	1104	1100	0.13 ± 0.00	0.38 ± 0.00	fat, citrus, green ^1^; waxy, aldehydic, citrus, fresh, green, lemon peel, cucumber, fatty ^2^
28	1-Octen-3-yl acetate *	1114	1112	0.05 ± 0.00		fresh, green, herbal, lavender, fruity, oily ^2^
29	3-Octanol acetate *	1125	1123	0.06 ± 0.00		fresh, bergamot, woody, green, grapefruit, rose, apple, minty ^2^
30	α-Campholenal	1127	1126		0.35 ± 0.02	herbal, green, woody, amber, leafy ^2^
31	*allo*-Ocimene	1131	1132	0.05 ± 0.00	0.08 ± 0.01	sweet, floral, nut, skin, peppery, herbal, tropical ^2^
32	(*2E*)-Nonen-1-al	1161	1161	0.08 ± 0.00	1.04 ± 0.01	cucumber, fat, green ^1^; fatty, green, cucumber, aldehydic, citrus ^2^
33	Terpinen-4-ol	1177	1177	tr		turpentine, nutmeg, must ^1^; woody, mentholic, citrus, terpenic, spicy ^2^
34	Naphthalene *	1181	1181	tr		tar ^1^
35	α-Terpineol	1190	1188	0.05 ± 0.00	0.06 ± 0.01	oil, anise, mint ^1^; pine, woody, resinous, cooling, lemon, lime, citrus, floral ^2^
36	Myrtenol	1195	1195		0.17 ± 0.01	woody, pine, balsamic, sweet, minty, medicinal ^2^
37	*p*-Cymen-9-ol *	1208	1205	tr		
38	Octanol acetate *	1210	1213		0.12 ± 0.00	green, earthy, mushroom, herbal, waxy, fruity, apple ^2^
39	(*E*)-Carveol	1214	1216	tr		caraway, solvent ^1^
40	Thymol methyl ether	1237	1235	tr		woody, smoky, burnt ^2^
41	Geraniol	1257	1252	tr		rose, geranium ^1^; sweet, floral, fruity, rose, waxy, citrus ^2^
42	Linalool acetate	1259	1257	0.14 ± 0.00		sweet, fruit ^1^; sweet, green, floral, spicy, clean, woody, terpenic, citrus ^2^
43	(*E*)-Myrtanol *	1260	1261		tr	
44	(*2E*)-Decenal	1263	1263	0.11 ± 0.00	0.47 ± 0.01	orange, tallow ^1^; waxy, fatty, earthy, green, cilantro, mushroom, aldehydic, fried, chicken, fat, tallow ^2^
45	Nonanoic acid	1271	1269		0.07 ± 0.01	green, fat ^1^; waxy, dirty, cheesy, dairy ^2^
46	(*3Z*)-Hexenyl valerate *	1282	1281	tr		green, fruity, apple, pear, kiwi, banana, unripe banana, tropical ^2^
47	Bornyl acetate	1285	1283		0.55 ± 0.01	woody, camphoreous, mentholic, cedar, woody, spicy ^2^
48	Dihydroedulan II	1288	1284	0.26 ± 0.01		
49	Dihydroedulan I	1293	1292	0.41 ± 0.00		
50	(*2E*,*4E*)-Decadienal	1316	1316		0.06 ± 0.00	fried, wax, fat ^1^; oily, cucumber, melon, citrus, pumpkin, nutty ^2^
51	δ-Elemene *	1339	1338	0.77 ± 0.00	0.44 ± 0.03	wood ^1^; sweet, herbal, lavender, woody ^2^
52	α-Cubebene	1351	1351	tr		citrus, fruit ^1^; herbal, waxy ^2^
53	Cyclosativene *	1368	1371	tr	0.09 ± 0.01	
54	α-Copaene	1377	1376	0.44 ± 0.01		wood, spice ^1^; woody, spicy, honey ^2^
55	(*E*)-Myrtanol acetate *	1385	1386		0.16 ± 0.01	
56	β-Bourbonene	1385	1388	0.50 ± 0.00		herb ^1^; herbal, woody, floral, balsamic ^2^
57	β-Cubebene	1388	1388	0.12 ± 0.00	0.09 ± 0.01	citrus, fruit ^1^; herbal, waxy, citrus fruity radish ^2^
58	β-Elemene	1393	1390	3.71 ± 0.03	0.65 ± 0.01	herb, wax, fresh ^1^; herbal, waxy fresh ^2^
59	α-Funebrene *	1401	1402		tr	
60	dihydro-α-Ionone *	1404	1406	0.10 ± 0.00		woody, floral, berry, orris, powdery, violet, raspberry, fruity ^2^
61	α-Barbatene *	1408	1407		1.42 ± 0.00	
62	α-Cedrene *	1413	1411	tr	0.24 ± 0.00	woody, cedar, sweet, fresh ^2^
63	α-Santalene *	1415	1417		0.06 ± 0.01	woody ^2^
64	(*E*)-Caryophyllene	1420	1419	5.29 ± 0.02	4.51 ± 0.03	wood, spice ^1^; sweet, woody, spicy, clove, dry ^2^
65	(*Z*)-Thujopsene *	1430	1431		0.11 ± 0.01	
66	β-Gurjunene	1431	1432	0.33 ± 0.00	0.58 ± 0.01	
67	β-Copaene	1432	1433	tr	0.06 ± 0.00	wood, spice ^1^
68	γ-Elemene	1437	1436	0.18 ± 0.00	1.06 ± 0.03	green, wood, oil ^1, 2^
69	Aromadendrene *	1440	1441	tr		wood ^1^
70	β-Barbatene *	1442	1442		4.26 ± 0.04	
71	(*Z*)-β-Farnesene	1445	1442	0.15 ± 0.00	tr	citrus, green ^1, 2^
72	(*E*)-Muurola-3,5-diene *	1449	1453	0.15 ± 0.00		
73	α-Humulene	1454	1454	1.34 ± 0.00	0.38 ± 0.01	wood ^1, 2^
74	(*E*)-β-Farnesene	1460	1456	4.83 ± 0.01	2.32 ± 0.03	wood, citrus, sweet ^1^; woody, citrus, herbal, sweet ^2^
75	Sesquisabinene *	1464	1459	0.07 ± 0.00		
76	9-*epi-*(*E*)-Caryophyllene *	1467	1466	tr		
77	β-Acoradiene *	1468	1470		3.77 ± 0.06	
78	γ-Gurjunene *	1475	1477		0.23 ± 0.01	musty ^2^
79	β-Chamigrene *	1477	1477		0.49 ± 0.01	
80	γ-Muurolene	1479	1479	0.43 ± 0.01		herb, wood, spice ^1^; herbal, woody, spicy ^2^
81	Germacrene D	1483	1481	17.53 ± 0.13	1.80 ± 0.01	wood, spice ^1^; woody, spicy ^2^
82	α-Curcumene	1483	1482		0.38 ± 0.01	herb ^1^; herbal ^2^
83	(*E*)-β-Ionone	1487	1488	0.07 ± 0.00		seaweed, violet, flower, raspberry ^1^; sweet, fruity, woody, berry, floral, seedy ^2^
84	β-Selinene *	1492	1490	0.38 ± 0.01	0.18 ± 0.00	herb ^1^; herbal ^2^
85	(*E*)-α-Bergamotene	1496	1494 ^D^	11.75 ± 0.02		wood, warm, tea ^1, 2^
86	Bicyclogermacrene	1497	1500		3.33 ± 0.01	green, wood ^1^; green, woody, weedy ^2^
87	α-Muurolene	1500	1500	0.21 ± 0.00	0.41 ± 0.00	wood ^1^
88	α-Chamigrene *	1502	1503		0.23 ± 0.01	
89	Cuparene *	1505	1504		0.89 ± 0.01	
90	Germacrene A	1505	1505	0.60 ± 0.00		
91	β-Bisabolene	1509	1505		1.66 ± 0.05	balsamic ^1^; balsamic, woody ^2^
92	(*E*,*E*)-α-Farnesene	1510	1505	7.23 ± 0.03	1.01 ± 0.01	wood, sweet ^1^; woody, green, vegetable, floral, herbal, citrus ^2^
93	γ-Cadinene	1515	1513	0.19 ± 0.01		wood ^1^; herbal, woody ^2^
94	δ-Cadinene	1517	1519	0.70 ± 0.02		thyme, medicine, wood ^1^; thyme, herbal, woody, dry ^2^
95	β-Bazzanene *	1520	1520	tr	4.10 ± 0.32	
96	Myristicin *	1522	1520		0.61 ± 0.01	spice, warm, balsamic ^1^; spicy, warm, balsamic, woody ^2^
97	(*E*)-Calamenene *	1522	1522	tr		herb, spice ^1, 2^
98	β-Sesquiphellandrene	1525	1522	1.51 ± 0.01	0.49 ± 0.01	wood ^1^; herbal, fruity, woody ^2^
99	(*Z*)-Nerolidol *	1534	1532	0.25 ± 0.01	0.68 ± 0.02	wax ^1^; waxy, floral ^2^
100	α-Cadinene *	1539	1538	0.06 ± 0.01		woody, dry ^2^
101	(*E*)-α-Bisabolene *	1545	1544	0.09 ± 0.01		balsamic, spicy, floral ^2^
102	α-Calacorene	1555	1545	0.16 ± 0.03		woody ^2^
103	Germacrene B	1558	1561	0.68 ± 0.01	4.59±0.03	wood, earth, spice ^1^; woody, earthy, spicy ^2^
104	(*E*)-Nerolidol	1565	1563	0.23 ± 0.01		wood, flower, wax ^1^; floral, green, citrus, woody, waxy ^2^
105	(-)-Spathulenol *	1576	1578	0.58 ± 0.00	0.12 ± 0.01	honey ^2^
106	Spathulenol	1578	1578	1.39 ± 0.01	2.94 ± 0.04	herb, fruit ^1^; earthy, herbal, fruity ^2^
107	Caryophyllene oxide	1583	1583	1.74 ± 0.02	1.35 ± 0.02	herb, sweet, spice ^1^; sweet, fresh, dry, woody, spicy ^2^
108	*allo*-Hedycaryol *	1587	1589	0.06 ± 0.00	0.19 ± 0.00	
109	Salvial-4(14)-en-1-one	1590	1594	0.10 ± 0.01	0.15 ± 0.01	
110	Cedrol *	1599	1596	0.30 ± 0.01		cedarwood, woody, dry, sweet ^2^
111	Widdrol	1602	1599	0.05 ± 0.01		
112	Santalol *	1606	1617	0.50 ± 0.03		sweet, sandalwood, woody ^2^
113	Humulene epoxide II	1609	1608	0.28 ± 0.01	0.08 ± 0.02	herbal ^2^
114	β-Atlantol *	1612	1608	0.29 ± 0.01		
115	γ-Eudesmol *	1630	1632	0.92 ± 0.01	0.11 ± 0.01	waxy, sweet ^2^
116	*epi*-α-Cadinol	1639	1640	0.43 ± 0.02	0.49 ± 0.00	
117	*t*-Muurolol	1647	1642	0.42 ± 0.00	0.12 ± 0.01	herbal, spicy, honey ^2^
118	α-Muurolol *	1643	1646	0.17 ± 0.01		balsamic, earthy ^2^
119	β-Eudesmol	1651	1650	0.14 ± 0.01		herbal, honey ^2^
120	α-Cadinol	1655	1654	1.70 ± 0.01	0.42 ± 0.01	herb, wood ^1^
121	*neo*-Intermedeol *	1661	1660	0.42 ± 0.01	0.11 ± 0.01	
122	14-hydroxy-(*Z*)-Caryophyllene *	1670	1667	0.35 ± 0.01	0.06 ± 0.01	
123	(*Z*)-α-Santalol *	1673	1675	0.56 ± 0.00	0.52 ± 0.10	woody, sandalwood ^2^
124	Apiole *	1680	1678	0.09 ± 0.00		woody, spicy ^2^
125	Elemol acetate *	1683	1680	0.13 ± 0.00	0.31 ± 0.09	
126	α-Bisabolol *	1686	1685		0.64 ± 0.16	floral, peppery, balsamic, clean ^2^
127	Germacra-4(15),5,10(14)-trien-1-α-ol *	1686	1686	0.21 ± 0.00		
128	(*Z*)-α-trans-Bergamotol *	1689	1690	1.09 ± 0.08		
129	Eudesm-7(11)-en-4-ol	1708	1700	0.07 ± 0.00		
130	14-hydroxy-α-Humulene *	1713	1714	0.05 ± 0.00	0.21 ± 0.01	
131	(*E*)-Nerolidyl acetate *	1717	1717	0.07 ± 0.00		fresh, sweet, citrus, waxy, freesia, woody ^2^
132	(*2E*,*6E*)-Farnesol	1737	1743	0.05 ± 0.01		muguet ^1^; muguet, floral, sweet, lily, waxy ^2^
133	β-Acoradienol *	1765	1763	0.06 ± 0.00	0.16 ± 0.01	
134	Hexahydrofarnesyl acetone	1846	1847	0.07 ± 0.00		oily, herbal, jasmine, celery, woody ^2^
135	Pentadecanoic acid *	1862	1862	tr	0.17 ± 0.01	waxy ^2^
136	Isophytol *	1948	1947	4.06 ± 0.01		floral, herbal, green ^2^
137	*n*-Hexadecanoic acid	1963	1960	0.05 ± 0.00	4.89 ± 0.06	waxy, fatty ^2^
138	(*Z*)-Falcarinol	2035	2036	0.57 ± 0.01	4.60 ± 0.01	
139	(*E*)-Phytol	2113	2122	1.15 ± 0.06		flower1; floral, balsamic, powdery, waxy ^2^
140	Linoleic acid *	2132	2133		1.37 ± 0.11	faint fatty ^2^
141	α-Linolenic acid *	2139	2143		0.18 ± 0.01	faint fatty ^2^
142	(*E*)-Phytol acetate *	2219	2218	tr	0.08 ± 0.01	waxy, floral, fruity, green, orchid, oily, balsamic ^2^
143	*n*-Pentacosane	2500	2500	tr		
144	Heptacosane *	2700	2700	tr		
	Total identified, %			117/99.39	88/99.24	

^#^ Compounds are listed in order of their elution from the nonpolar Rxi-5 MS capillary column. ^A^ An identification based on GC–MS spectra based on comparison with NIST 08 library standard reference database and calculated KI with those reported in Adams, NIST, PubChem, and Chemspider databases. ^B^ Kováts retention indices calculated against C_8_–C_32_ *n*-alkanes on nonpolar Rxi-5 MS column. ^C^ Kováts retention indices on nonpolar DB-5 column reported in the literature [25] or NIST (https://webbook.nist.gov (accessed on 10 June 2025)), PubChem (https://pubchem.ncbi.nlm.nih.gov (accessed on 10 June 2025)), and Chemspider (https://www.chemspider.com (accessed on 10 June 2025)) databases. ^D^ Kováts retention index on a nonpolar ZB-5 column reported in the literature [2]. ^E^ Relative percentage values are means of three determinations with a RSD%. ^F^ tr—trace (≤0.04%). * Compounds not reported in *Aegopodium podagraria* previously. ^1^ Odour description from https://www.flavornet.org (accessed on 25 September 2025). ^2^ Odour description from https://www.thegoodscentscompany.com (accessed on 25 September 2025).

Sixty-nine volatiles were identified in the EOs of the leaves and stems of *A. podagraria* growing wild in Estonia. The major EO components were α-pinene (6.6%, 15.7%), β-pinene (11.1%, 29.4%), limonene (8.2%, 18.4%), γ-terpinene (8.2%, 15.5%), germacrene D (15.6%, 1.5%), and (*E*)-α-bergamotene (4.8%, 0.4%) in EOs obtained from leaves and stems, respectively [2]. A different content of terpene compounds was reported in *A. podagraria* from the Iglinskii region (Bashkortostan), where bicyclic monoterpene sabinene comprised 62.9% of total volatiles [17]. Volatile compounds from the flowers, buds, and leaves of *Aegopodium podagraria* from southern Sweden included monoterpenes limonene (33%), β-pinene (26%), γ-terpinene (11%), and the sesquiterpene γ-cadinene (10%) [26]. Of the thirty-six compounds identified in goutweed EO from the Kharkiv region (Ukraine), sesquiterpenes and monoterpenes amounted to 90.7% and 6.8%, respectively, of the total; (*E*)-β-farnesene (43.94%), α-bergamotene (15.32%), (*E*,*E*)-α-farnesene (8.84%), and 1,5,9,9-tetramethyl-1,4,7-cycloundecatrien (5.51%) were the principal components [5].

Fifty-eight volatile compounds with concentrations > 100 ppm were identified in the EO from *A. podagraria* (voucher no. AP6031) from Kopaonik (Central Balkan), accounting for 96.1% of the total oil. The major components were (+)-α-pinene (13.3%), *p*-cymene (8.8%), (+)-limonene (9.4%), (*Z*)-β-ocimene (5.2%), germacrene D (4.7%), (+)-spathulenol (4.4%), α-thujene (4.2%), and perilaldehyde (4.1%) [16].

### 2.2. Antioxidant Potential of A. podagraria Extracts

Some previous studies have demonstrated that the *A. podagraria* plant may be a promising source for the development of drugs, dietary supplements, functional foods, and/or cosmetics [1,5,8,10,12,14,20,27]. The phytochemical composition and antioxidant properties of plant preparations, among other factors, depend on the morphological part that was used and the extraction techniques; it is vital to select favourable processing conditions. The antioxidant activity of goutweed has been previously screened in vitro [12,27,28]; however, studies on its antioxidant activity, especially of extracts obtained with solvents of different polarities, are relatively scarce. PLE was not applied to this plant. Consequently, to receive more comprehensive data, in our study, conventional hydrodistillation techniques were used to recover EOs and boiling water extracts, which were generated as hydrodistillation residues (by-products). At the same time, PLE with highly polar methanol and medium polarity acetone was applied to the dried leaves and roots. The yields of EO isolated from many aromatic plants are typically lower than 1%; therefore, valorization of the non-volatile distillation residues may be an option for increasing the sustainability and effectiveness of EO-bearing plants processing, leading to the development of zero-waste industrial biorefining technologies for the complete recovery of higher-value substances. For this reason, the liquid fraction after hydro-distillation was separated from the solids, then freeze-dried to obtain WE containing boiling-water-soluble, non-volatile constituents, including polyphenolic antioxidants. Following the recommendation of experts [29], several methods (TPC, DPPH^•^, and ABTS^•+^ scavenging, and ORAC) were used to provide a more comprehensive in vitro evaluation of the antioxidant potential of the biomaterials.

The extraction yields, TPC, and antioxidant capacity of the different leaf and root extracts of goutweed and GLEO are presented in Table 2. Higher yields of WE and AE extracts were obtained from goutweed leaves than from roots, except for GRME; the lowest extract yields were obtained with acetone. The yields of extracts followed the following order: GLWE > GRME > GRWE > GLME > GLAE > GRAE (Table 2).

The TPC values varied from 12.88 mg (GRME) to 62.12 mg GAE/g edw (GLWE). The TPC was more than twice as high in the extracts obtained from the leaves compared to those of the roots or GLEO (Table 2).

Nizioł-Łukaszewska et al. [12] reported that at the highest tested concentration, 10%, the content of TPC and flavonoids in their water–glycerin extract was 134 and 58 mg GA/g, and suggested that these could be primarily responsible for the antioxidant activity of the Apiaceae family’s extracts. The differences in TPC values could be explained by the efficiency of phenolic compound isolation from the plant, which depends on the sample matrix, molecular structure, concentration, and polarity. Due to the presence of hydroxyl groups and attached saccharides, most polyphenolic antioxidants are polar. The majority of the phenolic compounds present in *A. podagraria* leaves were dissolved in a protic solvent (boiling water) during the hydrodistillation procedure. However, the highest TPC from the roots was obtained with acetone, which differences in the composition of secondary metabolites in the roots and leaves could explain.

Pearson’s correlation between the TPC and antioxidant activity was calculated. The correlation analysis demonstrated that total phenolic content (TPC) is a major contributor to antioxidant capacity, particularly in assays that rely on hydrogen atom transfer and radical scavenging mechanisms. Strong positive correlations were observed between TPC and ORAC (r = 0.843, *p* = 0.035) and DPPH scavenging activity (r = 0.777, *p* = 0.040), both statistically significant, indicating that phenolic compounds substantially enhance antioxidant performance in these systems. Conversely, a strong negative correlation was found between TPC and DPPH EC_50_ (r = −0.841, *p* = 0.036), confirming that extracts with higher phenolic content require lower concentrations to achieve 50% radical inhibition. In contrast, ABTS assay results exhibited weaker associations with TPC (r = 0.627, *p* = 0.132 for ABTS values; r = −0.301, *p* = 0.562 for ABTS EC_50_), neither of which reached statistical significance. These findings suggest that while phenolic compounds are key determinants of antioxidant activity, the strength of their contribution varies across assay systems, with ORAC and DPPH being more sensitive indicators than ABTS. Additionally, it was observed that DPPH^•^ scavenging was strongly correlated (r = 0.947, *p* = 0.001) with the ABTS^•+^ assay results.

**Figure 2:**
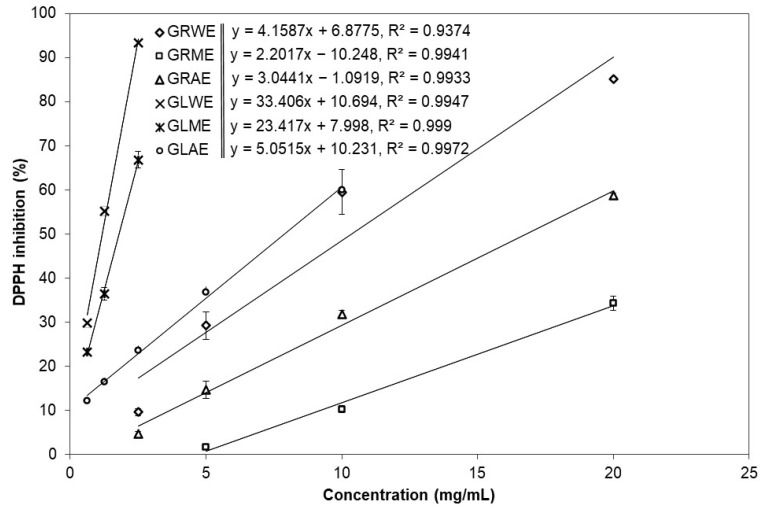
Effects of different concentrations of root water (GRWE), root methanol (GRME), root acetone (GRAE), leaf water (GLWE), leaf methanol (GLME), leaf acetone (GLAE) extracts of *Aegopodium podagraria* on DPPH^•^ scavenging capacity.

**Figure 3:**
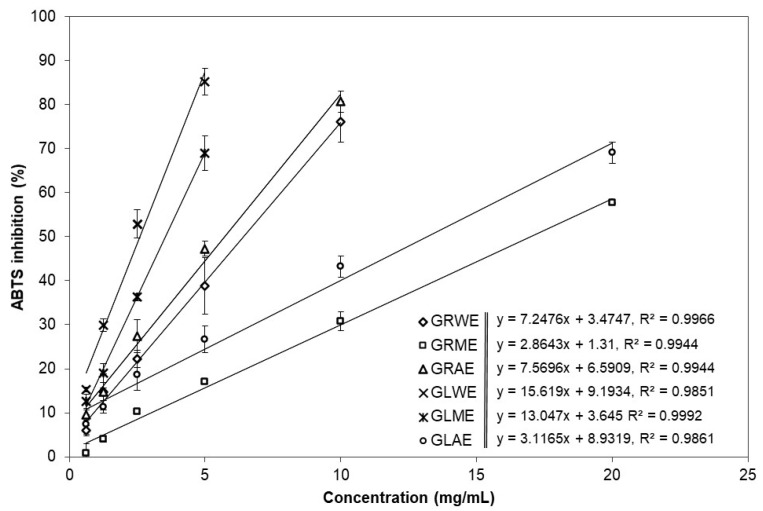
Effects of different concentrations of root water (GRWE), root methanol (GRME), root acetone (GRAE), leaf water (GLWE), leaf methanol (GLME), and leaf acetone (GLAE) extracts of *Aegopodium podagraria* on ABTS^+•^ scavenging capacity.

The chemical principles of the applied TPC, DPPH^•^, and ABTS^•+^ assays are somewhat similar; they are based on a single electron (SET) and or hydrogen atom (HAT) transfer [29]; however, the DPPH^•^ scavenging assay is more applicable for hydrophilic antioxidants, while the ABTS^•+^ decolourisation assay is suitable for both lipophilic and hydrophilic antioxidants. The extracts of *A. podagraria* ‘s leaves were more potent antioxidants than those of the roots. EC_50_ values representing the concentration required to reduce 50% of the radicals were estimated from the curves shown in Figure 2 and Figure 3, which depict linear dose–response relationships between extract concentration and radical scavenging activity in the DPPH^•^ and ABTS^•+^ assays, respectively. The individual regression lines for each extract illustrate apparent differences in their antioxidant effectiveness, and were used to calculate the corresponding EC_50_ values presented in Table 2. The steepness (slope) of these lines indicates how rapidly radical scavenging increases with increasing concentration; thus, steeper lines correspond to more effective extracts at a given concentration. In both assays, the curves of leaf extracts generally lie above those of root extracts at the same concentration range, visually confirming the higher antioxidant potency of *A. podagraria* leaves compared with its roots. Based on the DPPH^•^ and ABTS^•+^ assays, and the EC_50_ and µM TE values, GLWE and GLME were the most potent antioxidants. The weakest antioxidants were GRME and GLEO, as measured by the ABTS^•+^ scavenging assay, while the lowest antioxidant activity, in GLAE, was determined by the ABTS^•+^ assay (Table 2).

In general, polar solvents—water and methanol—were more effective than acetone for extracting natural antioxidants from the goutweed plant; these solvents yielded the highest extract yields and possessed the best antioxidant properties. Nizioł-Łukaszewska et al. [12] reported that the EC_50_ of goutweed water–glycerin extract obtained by ultrasound-assisted extraction was 38 mg/mL in the DPPH^•^ scavenging assay. Flieger et al. [28] optimized the DPPH-HPLC-DAD method for antioxidant activity determination in ethanol extracts of *A. podagraria*—the scavenging capacity was expressed by the percentage of peak inhibition and the IC_50_ parameters—and concluded that an extract prepared from dry plants in an ultrasonic bath exhibited the highest antioxidant potential (IC_50_ = 64.74 ± 0.22 µL/mL). Valyova et al. [30] reported that the ethanol extract of *A. podagraria*‘s aerial parts demonstrated the highest antioxidant capacity: Its IC_50_ value in a DPPH^•^ assay was 66.135 ± 1.6 μg/mL, and in ABTS^•+^ was 73.9 ± 8.7 μg/mL.

**Figure 4:**
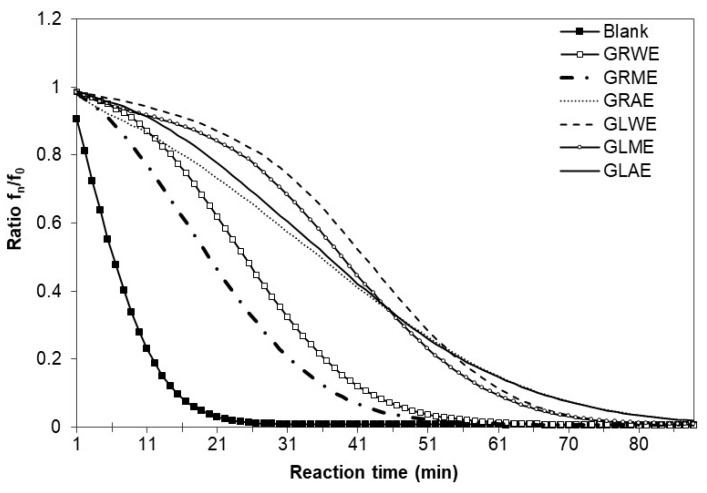
The curves obtained using various extracts in the ORAC assay: *f_0_* is the initial fluorescence, and *f_n_* is the fluorescence at time *n*.

The ORAC assay is considered most closely related to oxidation in biological systems [31]. ORAC values were significantly higher than those determined by other methods (Table 2). The ORAC assay is based on radical chain-breaking by HAT and evaluates the inhibition of oxidation induced by peroxyl radicals. It is believable that the ORAC assay is more relevant to the expected antioxidant capacity of polyphenols in biological systems. ORAC values measured in our study varied from 484.2 ± 78.0 (GRME) to 1425 ± 56 µM TE/g edw (GLWE). In general, extracts with higher total phenolic content also exhibited higher ORAC values: the leaf water and methanol extracts (GLWE and GLME) combined the highest TPC with the strongest peroxyl radical-scavenging capacity, whereas the methanolic root extract (GRME), which contained the lowest amount of phenolics, showed the weakest ORAC response (Table 2). This relationship indicates that polyphenolic constituents—including hydroxycinnamic acids and flavonoids previously reported in *A. podagraria*—substantially contribute to the chain-breaking antioxidant activity measured by ORAC. Since peroxyl radicals are among the most abundant reactive oxygen species generated during lipid peroxidation in biological systems, the high ORAC values of goutweed leaf extracts suggest a relevant potential to protect biomolecules against oxidative damage and support their prospective use in nutraceutical and cosmeceutical formulations. The curves in Figure 4 represent the kinetics of oxygen absorbance capacity by the different extracts. These curves clearly indicate that qualitative and/or quantitative compositions of the antioxidants present in different *A. podagraria* leaf and root extracts are significantly different. Although the ORAC values were numerically higher than those obtained by DPPH^•^ and ABTS^•+^ assays, the absolute values of the other methods cannot be directly compared, because they rely on different radicals, reaction mechanisms, and endpoints; therefore, only the relative trends among the extracts were considered when interpreting the data.

## 3. Materials and Methods

### 3.1. Plant Material

*Aegopodium podagraria* L. (goutweed) was collected in 2022 in the Seniava district, Kaunas, Lithuania (54°50′45″ N of latitude, 23°54′39″ E of longitude, elevation altitude 84 m). The leaves were collected at the end of May–beginning of June (pre-flowering phase) and the roots at the end of October. The plant was dried at room temperature in a dark, ventilated room and stored in glass containers.

### 3.2. Chemicals and Solvents

2,2-Diphenyl-1-picrylhydrazyl (DPPH^•^, 95%), 2,2-azinobis-3 ethyl benzothiazoline-6-sulphonic acid (ABTS), 6-hydroxy-2,5,7,8-tetramethylchromane-2-carboxylic acid (Trolox, 97%), 2,2′-azobis(2-methylpropionamidine) dihydrochloride (97%), potassium persulfate (K_2_S_2_O_8_) (≥99%), sodium phosphate salts (99%), gallic acid (GA), Folin–Ciocalteu phenol reagent (2 M, 99%), anhydrous Na_2_CO_3_, NaCl, KCl, KH_2_PO_4_ were obtained from Sigma-Aldrich (St. Louis, MO, USA). Fluorescein sodium salt (FL) was obtained from Fluka Chemicals (Steinheim, Germany); the solvents acetone and methanol were obtained from Lachema (Brno, Czech Republic); and ethanol was obtained from JSC Stumbras (Kaunas, Lithuania). A standard mixture of C_8_-C_32_ *n*-alkanes (Supelco Analytical, Bellefonte, PA, USA) was used to determine Kováts retention indices (KI).

### 3.3. Isolation of Essential Oils (EOs) and Preparation of Goutweed Extracts

Dried leaves and roots were ground in an ultra-centrifugal mill ZM200 (Retsch, Haan, Germany) using a 1.0 mm sieve. The EOs were isolated from 100 g using hydro-distillation in a Clevenger-type apparatus (TECHNOSKLO s.r.o., Držkov, Czech Republic) for 3 h. After distillation, the liquid fraction residues were separated from the solids by filtration and freeze-dried in a Maxi Dry Lyo (Heto-Holton AIS, Allerod, Denmark), yielding the water extracts of the leaves (GLWE) and roots (GRWE). In addition, the ground leaves and roots were directly extracted with methanol and acetone using pressurized liquid extraction (PLE) on a Dionex ASE 350 system (Dionex, Sunnyvale, CA, USA). The ground roots or leaves (10 g) were mixed with 1 g of diatomaceous earth in 34 mL Dionex stainless-steel vessels. A three-cycle program for 5 min each was used for extraction (15 min total time) at 70 °C and 10 MPa [32], which represents compromise conditions widely used in the PLE of plant matrices, providing efficient extraction of medium- and high-polarity constituents while limiting the risk of thermal degradation of phenolic antioxidants. Three extraction cycles were applied to approach exhaustive extraction of the ground material without excessive solvent consumption or processing time. A flush volume of 100% and a 60s purge with nitrogen gas were set at the end of each extraction. The extracts were concentrated in a rotary vacuum evaporator Rotavapor® R-210 (BÜCHI Labortechnik AG, Flawil, Switzerland) at 40 °C and stored at −18 °C until analysis. All the extractions were performed in triplicate. The yield of EOs and extracts was expressed in % (*w*/*w*) of plant dry weight (pdw).

### 3.4. Gas Chromatography–Mass Spectrometry (GC-MS)

The EOs isolated from leaves and roots were diluted in pentane (5 μL/mL) and analyzed on a Shimadzu GC-2010 Plus gas chromatograph coupled to a GCMS-QP2010 Ultra Shimadzu mass selective detector (Shimadzu, Kyoto, Japan). The compounds were separated in a Rxi-5 MS (5% diphenyl, 95% dimethylpolysiloxane) capillary column, 30 m length, 0.25 mm i.d., 0.25 μm film thickness (Restek, Bellefonte, PA, USA) under the same method and conditions as reported by Baranauskienė et al. [21].

The components were identified by comparing their Kováts retention indices (KI) relative to C_8_–C_32_ *n*-alkanes with those provided in the literature [25] and by comparing their mass spectra (MS) with NIST standard reference database (NIST 08 library and NIST library search program version 2.0d). The mean values were calculated from triplicate injections. Identification was assumed when a good match of MS and KI was achieved.

### 3.5. Total Phenolic Content (TPC)

The Folin–Ciocalteu method with slight modifications [33] was used. A linear calibration curve was constructed by mixing 0.2 mL of gallic acid solutions in ethanol (0–0.5 mg/mL) with 2 mL of a diluted Folin–Ciocalteu reagent prepared with ultra-pure water (1:10) and 2 mL of a 7% Na_2_CO_3_ solution. The absorption was measured after 90 min at 765 nm on a Spectronic Genesys 8 spectrophotometer (Thermo Fisher Scientific, Rochester, NY, USA); a calibration curve was obtained with gallic acid solutions in ethanol over the concentration range 0–0.5 mg/mL (R^2^ = 0.9991). Similarly, 0.2 mL of 0.1–0.25% of WE in ultra-pure water, AE, ME in methanol, and distilled water as a blank were analyzed. Four replicate measurements were performed for each sample. The TPC was expressed in milligrams of gallic acid equivalents per gram of plant extract (mg GAE/g edw).

### 3.6. DPPH^•^ Scavenging Capacity

The DPPH scavenging activity was measured using the method by Brand-Williams et al. [34]. A quantity of 7.5 μL of extract (concentrations were in the range of 0.00625–2%) or Trolox solution per sample was mixed in microplate wells with 300 μL of a methanolic solution containing 60 μM DPPH^•^. A blank sample was made with methanol. Absorbance was measured in a microplate reader FLUOstar Omega (BMG Labtech, Durham, NC, USA) at 517 nm at every minute until the absorption curve reached a plateau (total 35 min). The Trolox standard in concentrations of 0.06–1 mM was used to estimate a standard curve. The final results were expressed as effective concentrations (EC_50_), which denote the extract concentration required to decrease the initial DPPH^•^ concentration in the reaction mixture by 50%, and as µM Trolox equivalents per gram of extract dry weight (µM TE/g edw)

### 3.7. ABTS^•+^ Scavenging Capacity

ABTS was measured according to the method by Re et al. [35]. The stock solution was prepared by dissolving two mM ABTS in 50 mL phosphate-buffered saline (pH 7.4). The ABTS^•+^ solution was prepared by reacting 50 mL of the ABTS stock solution with 200 μL of a 70 mM K_2_S_2_O_8_ solution, then incubating the mixture at room temperature in the dark for 15 h. The stock solution was then diluted with phosphate-buffered saline to a final absorbance of 0.70 (±0.02) at 734 nm. Spectrophotometric measurements were performed in 96-well microplates. A quantity of 3 μL of sample solution was mixed with 297 μL of the ABTS^•+^ solution, and the absorbance was measured in a microplate reader FLUOstar Omega (BMG Labtech, Durham, NC, USA). A 300 μL volume of PPB solution was used as a blank sample. The calibration curve was prepared using the Trolox standard in the concentration range of 0.6 to 2 mM. A sample concentration providing 50% inhibition of ABTS^•+^ (EC_50_) was determined from a graph of inhibition percentage versus extract concentration. The scavenging activity was also expressed as µM TE/g edw.

### 3.8. Oxygen Radical Absorbance Capacity

The rates of oxidative degradation of the fluorescent compound (fluorescein sodium salt) after exposure to an oxygen radical initiator, 2,2′-azobis(2-methylpropionamidine), were measured according to the method by Huang et al. [31], which represents the oxygen radical absorbance capacity (ORAC) of the extract. All solutions were prepared in 75 mM phosphate buffer at pH 7.4 (PBS). The measurements were carried out in 96-well black microplates. To each well, 25 μL of the sample was added, followed by 150 μL of fluorescein (96 mM). The mixture was incubated for 15 min at 37 °C in the built-in incubator. Afterward, 26 μL of 2,2′-azobis (2-methylpropionamidine) solution (240 mM) was manually added, and the microplate was shaken for 30 s. Fluorescence was recorded (λ_ex_ = 493 nm, λ_em_ = 515 nm) every 66 s for 90 min in the fluorimeter microplate reader FLUOstar Omega (BMG Labtech, Durham, NC, USA). Trolox solutions at concentrations of 10–200 μM were prepared for a standard calibration curve. The ORAC values were calculated from differences in the areas under the fluorescence decay curves between the blank, samples, and standards. The area under the fluorescence decay curve (AUC) was calculated as follows: AUC = 1 + sum (*f_1_*/*f_0_* + *f_2_*/*f_0_* + *f_3_*/*f_0_* + *f_4_*/*f_0_* + *f_5_*/*f_0_* + *f_6_*/*f_0_* + *f_7_*/*f_0_* + … + *f_n_*/*f_0_*), where AUC is the area under the FL decay curve (X). The *f_0_* is the fluorescence reading at time 0 (initial fluorescence), and *f_n_* is the fluorescence reading at time *n*. Net AUC (standard + sample): AUC - AUC blank, where Net AUC is the net area under the FL decay curve (X). Finally, ORAC values were expressed as µM TE/g edw.

### 3.9. Statistical Analysis

Mean values ± standard deviations of extract yields, antioxidant capacity values, and percentage contents of EO components were calculated from 3 to 4 replicate determinations using MS Excel 2010 software. Statistical comparisons among different groups presented in Table 2 were performed by one-way analysis of variance (ANOVA) using Statgraphics Centurion XVIII package (Statgraphics Technologies, Inc., The Plains, VA, USA). Tukey’s HSD was performed as a post hoc analysis at a 95% confidence level.

## 4. Conclusions

Our study significantly expanded knowledge of the composition of the essential oils (EOs) of *Aegopodium podagraria*: 58 compounds of the 144 reported from the hydro-distilled volatile oils of the leaves and roots were tentatively or positively identified for the first time. Sesquiterpenes germacrene D and (*E*)-β-bergamotene were the major volatiles in the leaf EO, while α-pinene, germacrene B, (*E*)-caryophyllene, hexadecanoic acid, and (*Z*)-falcarinol were dominant in the root EO. The study demonstrated that hydrodistillation residue can be valorized to produce water-soluble natural antioxidants. At the same time, pressurized liquid extraction with solvents of different polarities can yield two distinct products from dried leaves and roots. High-polarity water and methanol extracts from goutweed leaves exhibited the highest total phenolic content (TPC) and oxygen radical absorbance capacity (ORAC). Consequently, the plant biorefining process for producing valuable aromatic and antioxidant fractions on a laboratory scale may be considered a first step toward developing new industrial ingredients for nutraceuticals, cosmeceuticals, flavour/fragrance, and other products. Further research should focus on a more comprehensive evaluation of the extract’s composition and bioactivities of its individual constituents. At the same time, life-cycle and techno-economic assessments would be essential for upscaling and commercialization of the processes and products obtained.

## Figures and Tables

**Table 2:** The yield, total phenolic content, and antioxidant capacity of the extracts and essential oil of *Aegopodium podagraria*.

Extract	Yield (%)	TPC mg GAE/g edw	DPPH EC_50_ (mg/mL)	DPPH µM TE/g edw	ABTS EC_50_ (mg/mL)	ABTS µM TE/g edw	ORAC µM TE/g edw
GRWE	26.47 ± 0.60 ^c^	20.37 ± 0.15 ^c^	10.36 ± 0.49 ^c^	43.46 ± 2.11 ^d^	6.44 ± 0.20 ^b^	177.38 ± 5.53 ^c^	700.49 ± 62.74 ^b^
GRME	29.54 ± 1.45 ^d^	12.88 ± 0.24 ^a^	27.41 ± 1.04 ^f^ *	16.42 ± 0.61 ^b^	8.69 ± 0.35 ^d^	131.47 ± 5.19 ^b^	484.21 ± 78.00 ^a^
GRAE	3.53 ± 0.23 ^b^	25.30 ± 0.28 ^d^	16.78 ± 0.07 ^e^	26.79 ± 0.12 ^c^	5.73 ± 0.12 ^b^	199.01 ± 4.20 ^c^	1222.18 ± 53.75 ^c^
GLWE	37.02 ± 0.83 ^e^	62.12 ± 1.10 ^g^	1.18 ± 0.01 ^a^	382.16 ± 4.19 ^f^	2.45 ± 0.14 ^a^	467.64 ± 26.29 ^e^	1425.59 ± 56.42 ^c^
GLME	25.25 ± 1.45 ^c^	56.84 ± 1.05 ^f^	2.48 ± 0.11 ^b^	181.57 ± 7.65 ^e^	3.57 ± 0.25 ^a^	320.33 ± 22.04 ^d^	1293.06 ± 118.70 ^c^
GLAE	4.70 ± 0.30 ^b^	51.49 ± 0.84 ^e^	11.93 ± 0.31 ^d^	37.71 ± 0.97 ^d^	13.20 ± 0.62 ^c^	86.60 ± 4.24 ^a^	1285.91 ± 61.39 ^c^
GLEO	0.22 ± 0.01 ^a^	16.52 ± 0.23 ^b^	–	2.97 ± 0.69 ^a^	–	171.93 ± 5.95 ^c^	–

^a–g^ Different superscript letters within the same column indicate statistically significant differences between extracts (*p* < 0.05, one-way ANOVA, Tukey’s HSD). * This extract has not reached 50% inhibition at the highest applied concentration, and the EC_50_ value was extrapolated from the linear fit of three concentration points in Figure 2.

## Data Availability

Data available from the authors upon request.
